# Analgesic Effect of Low Dose Nefopam Hydrochloride after Arthroscopic Rotator Cuff Repair: A Randomized Controlled Trial

**DOI:** 10.3390/jcm8040553

**Published:** 2019-04-24

**Authors:** Hyun Jung Koh, Jin Joo, Yang-Soo Kim, Yu Jung Lee, Woojoo Yoo, Min Soo Lee, Hue Jung Park

**Affiliations:** 1 Department of Anesthesiology and Pain Medicine, Seoul St. Mary’s Hospital, College of Medicine, The Catholic University of Korea, Seoul 137-701, Korea; uglyko@hanmail.net (H.J.K.); jiyo1004@catholic.ac.kr (J.J.); dasaki7@gmail.com (Y.J.L.); yoo.astro@gmail.com (W.Y.); choidouble@naver.com (M.S.L.); 2 Department of Orthopedics, Seoul St. Mary’s Hospital, College of Medicine, The Catholic University of Korea, Seoul 137-701, Korea; kysoos@catholic.ac.kr

**Keywords:** rotator cuff, nefopam hydrochloride, interscalene block, postoperative pain, Visual Analog Scale (VAS), postoperative nausea and vomiting (PONV)

## Abstract

Arthroscopic rotator cuff repair causes acute postoperative hyperalgesia. Multimodal analgesia is preferable to opioid-based intravenous patient-controlled analgesia (IV-PCA) due to postoperative nausea and vomiting (PONV). We evaluated the effect of nefopam as a postoperative non-opioid analgesic after shoulder surgeries. A total of 180 adult patients were enrolled for arthroscopic rotator cuff repair. They were randomly assigned to nefopam (N) or control (C) groups and each group was reclassified according to the interscalene block (B) into NB, CB and NX, CX. Nefopam was applied at a constant dose intravenously during recovery. Pain scores were measured with a Visual Analogue Scale (VAS) before (T0), immediately after (T1), 30 min (T2) and 12 h (T3), 24 h (T4) and 48 h (T5) after surgery. There was no significant difference in demographic data. The overall VAS scores did not differ with regard to nefopam use, except for the NB group at T4 in intention to treat (ITT) analysis (*p <* 0.05). PONV occurred more frequently in the N group than in the C group (*p* < 0.05). Neither individual nor all risk factors were associated with PONV occurrence (*p* > 0.10). In conclusion, nefopam alone did not show a definite decrease in postoperative pain. It instead increased PONV regardless of risk factors.

## 1. Introduction

In general, even though postoperative pain after arthroscopic rotator cuff repair is less than that experienced in other types of orthopedic surgery (<4 in Visual Analog Scale (VAS)), pain control is required. Severe pain typically develops on the first postoperative day [1] and is observed in up to one-third of patients [2]. However, the application of opioid analgesics tends to be restricted in shoulder surgeries due to its side effects. Therefore, there needs to be a balance between the requirements for pain reduction and the opioid application [3,4]. Administering intermittent analgesics to avoid continuous opioid analgesia leads to discomfort and annoyance for both the patients and the medical team. Therefore, various analgesic strategies have been introduced to improve patient rehabilitation and postoperative outcomes [5]. In particular, if acute pain within the first postoperative 24 h is well controlled, patient satisfaction and long-term outcomes may be improved due to the reduction in shoulder motion during this period.

Interscalene block (ISB) definitely decreases postoperative pain. It is being more frequently used as a first-choice analgesic but it cannot be applied after all operations. Consequently, multimodal analgesic methods, such as oral agents or intravenous (IV) analgesics, are used instead [5]. In this case, controlling postoperative pain immediately after surgery is a most important factor in deciding the outcome. Even if ISB is performed, the pain reoccurs if the duration of the nerve block is short and additional analgesics are necessary. Relatively long periods of postoperative pain and insufficient analgesics result in the patients complaining. However, no pain management is able to completely relieve discomfort. Recently, there is growing interest in additional or more effective analgesics [6].

Nefopam hydrochloride (Acupan^®^ 30 mg, Acupan inj. 20 mg, Pharmbio Korea Co., Ltd. Seoul., Korea) is one of them. Its mechanism has not yet been described in detail. However, nefopam affects postoperative pain not by acting on direct opioid receptors but by affecting triple neurotransmitter uptake inhibitors, such as norepinephrine, serotonin and the dopamine receptor [7]. Furthermore, nefopam has an effect on hyperalgesia and central sensitization [8] through the NMDA (N-methyl-D-aspartate) receptor and plays a role as a preemptive analgesic [9,10]. Due to the specific nature of this drug, various studies have been conducted to analyze whether it can be used to control pain more easily than opioids [8,10,11]. According to Sinclair et al. [4], patients with orthopedic shoulder surgery had a six-fold increase in their risk of postoperative nausea and vomiting (PONV). Furthermore, opioid-based intravenous patient-controlled analgesia (IV-PCA) can aggravate the possibility of PONV. Thus, we tried to determine the effectiveness of nefopam, a non-opioid analgesic, for pain control after orthopedic shoulder surgery. Although it has been used in combination with other analgesic agents to improve its effectiveness, there is a lack of research focusing on the preemptive analgesic and postoperative anti-hyperalgesic effects of nefopam when given for a short term period before and after surgery, especially concerning arthroscopic shoulder operations.

Therefore, this study aimed to confirm the effect of nefopam as a preemptive analgesic or the anti-hyperalgesic effect when applied as a low dose single agent (not in a combination) in arthroscopic shoulder surgery.

## 2. Materials and Methods

### 2.1. Study Design

We conducted a randomized controlled trial of patients with a rotator cuff tear that was less than 2 cm. These patients had visited Seoul St. Mary’s hospital. Ethical approval for this study (Ethical Committee N° KC15OISI0446) was provided by the Ethical Committee IRB (Institute Research Board) and participants provided informed consent before being part of this study.

### 2.2. Participants

Patients with American Society of Anesthesiologists physical status (ASA-PS) I–II were enrolled in this study if they were scheduled for elective arthroscopic rotator cuff repair due to medium (1–3 cm) tear. The exclusion criteria included an age under 20 or over 70 years of age, severe renal or hepatic insufficiency, severe cardiac disease, seizures, epilepsy, taking monoamine oxidase inhibitors, glaucoma, cerebrovascular disease, allergies or hypersensitivity to nefopam. Patients who could not understand the visual analog scale (VAS) were also excluded. 

### 2.3. Randomization and Allocation

The enrolled patients were assigned into two groups according to a computer-generated randomized table of numbers. First, the groups were divided into those that were administered nefopam (N) and those who were not administered nefopam (C) before each group was divided into ISB (B) and not (X), respectively. That is, patients were divided into four groups, which included the following sub-division categories: only nefopam (NX), nefopam with ISB (NB), without nefopam and ISB (CX) and ISB without nefopam (CB). This random allocation process was conducted by two medical staffs who were not involved in the study outcome evaluations. They generated the random allocation sequence and were involved directly in drug preparation and ISB. In accordance with the orthopedic surgical method at our hospital, the operation was performed in two operating rooms. Furthermore, the orthopedic surgeon and medical staff members who assessed outcomes were blinded after being assigned to each group. Only the patients knew whether to use nefopam and ISB after being allocated according to the RCT (Randomized Controlled Trials) table.

### 2.4. Procedure and Intervention

The severity of pain was assessed using a VAS from 0 (no pain) to 10 (maximum, unimaginable pain). Significant analgesic efficacy was defined as VAS being reduced by 2-fold or 30% compared with the VAS immediately before the assessment [12,13,14]. All patients were given a Cox-2 inhibitor (Celebrex®, 200 mg, 1 C) as a premedication in ward. The N group took nefopam (30 mg) right before surgery in the pre-operating room. The operation was conducted by one surgeon using the same surgical methods. General anesthesia was provided through inhalation and a prophylactic antiemetic, ramosetron (0.3 mg), was given just before the surgery was over in all groups. ISB was given to NB and CB groups, with 0.25% ropivacaine (14 cc) and 1% lidocaine (6 cc) given to the nerve innervating the C (cervical spine) 5–6 levels in an ultrasound guided process. All N groups were given a continuous infusion of nefopam (20 mg) mixed with normal saline for 30 min at 100 mL/h as soon as they arrived without using other opioid analgesics in a post-anesthetic care unit (PACU). All groups were injected with rescue analgesics in the same way: fentanyl (25 mcg) up to four times (max. 100 mcg) every 10 min in the PACU. After being transferred to the ward, designated analgesics, such as 12.5 mg of meperidine (pethidine) and 50 mg of tridol, were applied when patients complained of pain (VAS > 4). VAS was measured six times: before surgery (T0), right after surgery (T1), 30 min (T2), 12 h (T3), 24 h (T4) and 48 h (T5) after surgery.

### 2.5. Outcomes

The primary outcomes were pain severity at T2 in the X subgroups (NX and CX) and at T4 in the B subgroups (NB and CB). The secondary outcomes were pain severity at the remaining time points (T0, T1, T3 and T5), the number of rescue drugs administered and the relationship between PONV events and nefopam. The risk factors for PONV (female, nonsmoking, motion sickness, PONV history and postoperative opioid use) were assessed using the Apfel score [15] and the total operative time was also evaluated.

### 2.6. Statistical Analysis

To assess the extent of the pain reduction due to nefopam in shoulder surgery, 75 patients were recruited in each group to provide a study that had a power of 80% and an effect size of 0.5 according to our previous pilot study. Data are presented as means ± standard deviations, medians and interquartile range or numbers, including percentages based on each variables’ characteristics. Wilcoxon rank-sum test was performed for continuous variables and Chi-square test or Fischer’s exact test was performed for categorical variables. The relationship between nefopam and baseline risk factors was analyzed by logistic regression. A *p*-value < 0.05 was considered to be statistically significant and statistical analyses were conducted using SAS Version 9.4 (SAS Institute Inc., Cary, NC, USA).

## 3. Results

### 3.1. Study Flow Chart

**Figure 1:**
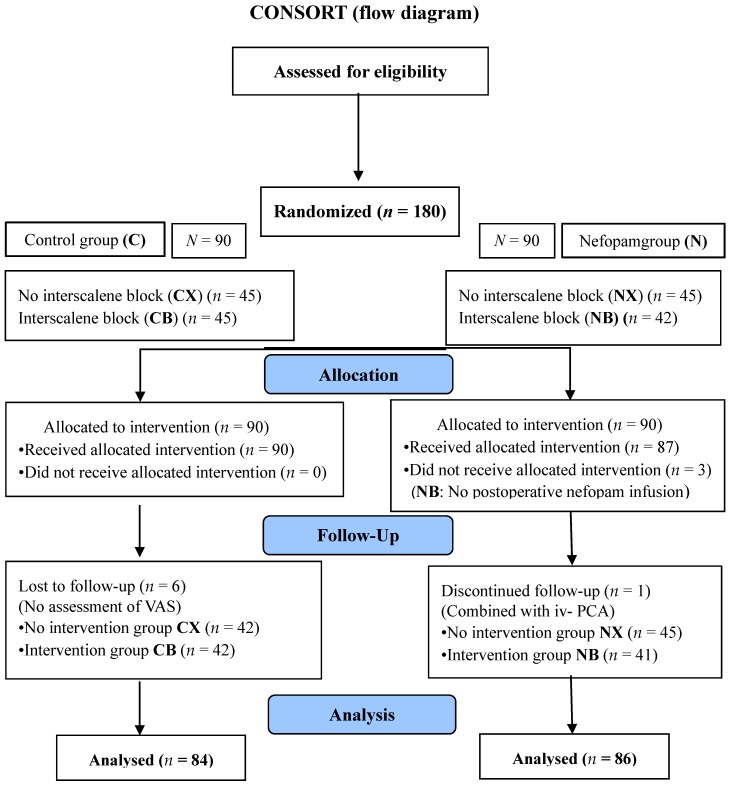
Flow diagram. N: groups administered nefopam, NX: only nefopam, NB: nefopam with ISB, CX: without nefopam and ISB, CB: ISB without nefopam.

A total of 180 patients were enrolled without exclusion. After allocation, three in the NB group (assigned to ISB) were excluded due to them missing a nefopam infusion after surgery. During the follow-up period, six in the C group (three in CB and three in CX) did not have their VAS checked and one in the NB group dropped out due to them needing an additional IV-PCA. Finally, among the remaining 170 patients, 84 in the C group and 86 in the group N were analyzed. A CONSORT (Consolidated Standards of Reporting Trials) diagram explaining the flow of participants is shown in Figure 1.

### 3.2. Demographic Data 

There were no differences in ASA-PS classification, age, sex, height, or weight between the groups (Table 1). The operative time (average, 87.6 ± 30.2 min) also showed no differences (*p* > 0.05). 

### 3.3. Association between VAS and Nefopam 

**Figure 2:**
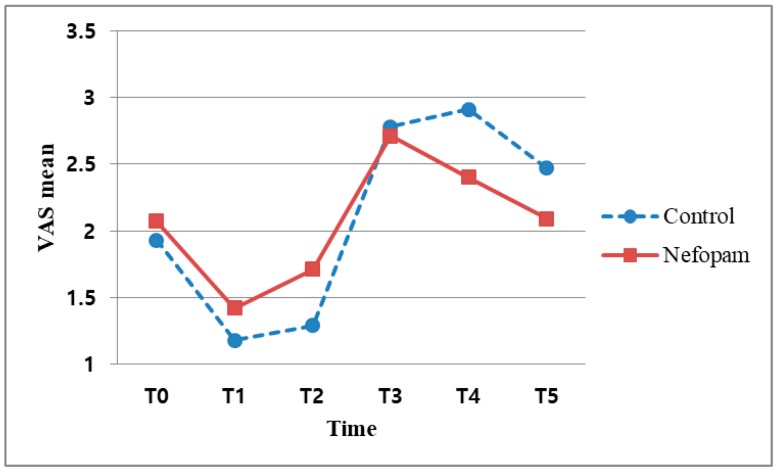
Association between VAS scores and time points. VAS: visual analogue scale; T0: before surgery, T1: immediately after surgery, T2: after surgery 30 min; T3: after surgery 12 h; T4: after surgery 24 h; and T5: after surgery 48 h.

The median VAS score was 3 or less in both the N and C subgroups regardless of ISB. There were no significant differences in the VAS score between the N and C groups regardless of ISB (*p* > 0.1) (Figure 2). The maximum VAS was 4 in the B subgroup and 7 in the X subgroup (Table 2). 

There was no significant difference in VAS between the groups according to sex (Table 3).

### 3.4. Difference in VAS between the Nefopam Group (N) and the Control Group (C) Regarding Block (B v.s. X) 

**Figure 3:**
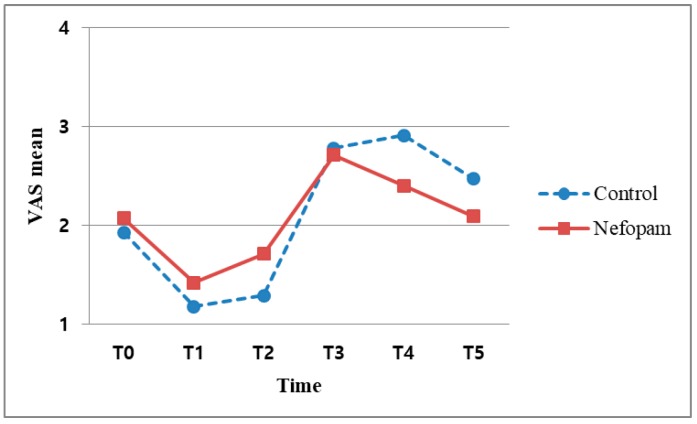
Association between VAS scores and time points without interscalene block.

**Figure 4:**
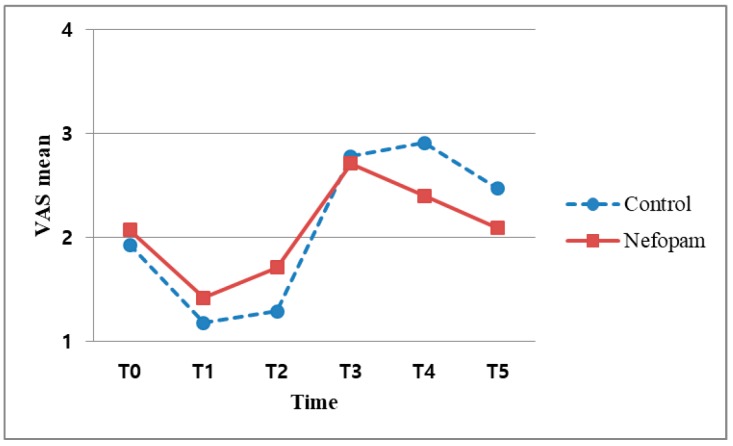
Association between VAS scores and time points after interscalene block.

The median VAS score was 5 or less in both the B and X subgroups When the VAS was evaluated at T3, there was no significant difference in the VAS scores between the NX and CX subgroups (*p* = 0.058). However, this *p*-value nearly reached statistical significance. In the per-protocol (PP) analysis, the *p*-values were also found to be significant (*p* = 0.037) (Table 4, Figure 3). In other words, there was no difference between the N and C groups without block at the 5% significance level, whereas a difference existed at the 1% significance level. In contrast, the VAS scores at T4 differed significantly between the NB and CB subgroups (*p* = 0.03) (Table 4, Figure 4). After an ISB, postoperative pain usually does not develop within 24 h, making it difficult to confirm the analgesic efficacy of nefopam during the acute postoperative period (Table 4).

### 3.5. Association between Nefopam and Total Number of Rescued Drugs 

There were no significant differences between the N and C groups in the total number of rescue drugs administered for postoperative pain (*p* = 0.187) (Table 5).

### 3.6. Association between PONV and the Presence of PONV Risk Factors 

**Figure 5:**
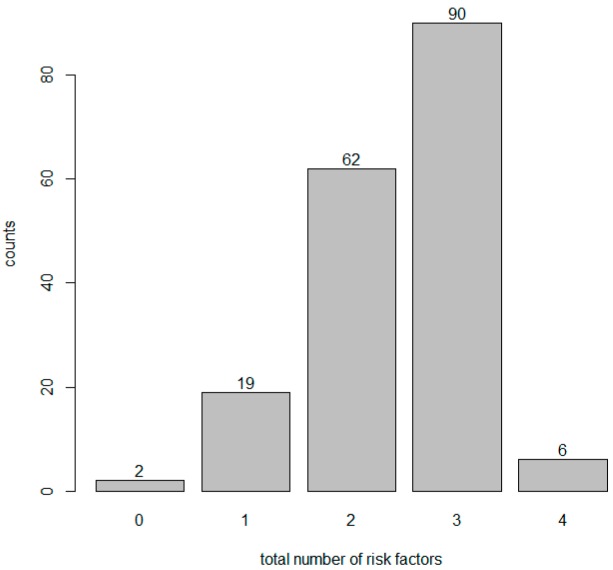
Patient distribution with respect to the total number of risk factors.

There was no relation between the occurrence of PONV and the presence of individual and all PONV risk factors (Table 6). Receiving nefopam (vs. control) had no effect on each PONV risk factor regardless of PONV events and the presence of each PONV risk factor (Table 7). In contrast, PONV occurred more frequently in the N group than in the C group (*p* = 0.023, Table 8) although more than 50% of patients had more than three risk factors (Figure 5). The total number of risk factors had no relationship with PONV occurrence (Table 9).

## 4. Discussion

After arthroscopic rotator cuff repair, nefopam hydrochloride was not able to reduce acute postoperative hyperalgesia when applied once preoperatively as a single agent. Furthermore, early postoperative nefopam infusion did not show a definite analgesic effect. In contrast to previous studies [1,16,17], we could not validate the postoperative analgesic efficacy of nefopam administered immediately after surgery. In particular, VAS scores measured at specific time points after surgery showed differences with respect to ISB application (B vs. X subgroups) from T0 to T4 but the difference was minimal at T5 (48 h). From this result, we concluded that the pain was the most severe during the first postoperative day (<24 h) [2], with this being the most important period for pain management. In other words, the level of pain can be an indicator of the postoperative outcome in arthroscopic shoulder surgery. The efficacy of nefopam had been previously demonstrated when it was administered as a combination analgesic. However, it is difficult to confirm the effect of nefopam as a single drug based on this study, which involved a short period of time. In a randomized controlled trial (RCT) study, the results are interpreted using an intention-to-treat (ITT) analysis. In the X subgroups (patients with no nerve block), there was no significant difference in the intention to treat (ITT) analysis of T3 (12 h) at the 5% significance level (*p* = 0.058), although the Per Protocol (PP) analysis did indicate significant differences (*p* = 0.037) between N and C groups. This result was significant at the 1% level but not the 5% level. Based on this analysis, we recommend that further studies should focus on the early postoperative analgesic effect of nefopam. Similar to previous studies [18], our results support the conclusion that this preoperative oral analgesic lacks demonstrated drug efficacy. In addition, considering that most operative durations were less than 90 min, this surgery was too short for the identification of a postoperative anti-hyperalgesic effect. Our result of nefopam not showing effectiveness is consistent with previous research [19]. Patients who received a continuous nefopam infusion during the perioperative and postoperative periods exhibited more marked results. The time to occurrence of postoperative pain differs depending on the availability of ISB. Specifically, in the case of a bolus injection of local anesthetic, the pain usually started between 12 and 24 h, which is also when the block effect subsided. A significant difference in the VAS scores was also observed at T4 (24 h) in the patients who underwent ISB with nefopam (NB subgroup; *p* = 0.03), suggesting a continuous analgesic effect of nefopam postoperatively (Figure 4). Based on the previous studies reporting that arthroscopic surgical pain was not excessively severe, we could not confirm a comparative analgesic effect at T5 (48 h). This result might not support the finding of a previous study [16], which reported a decrease in long-term pain hypersensitivity after a single nefopam administration. On the other hand, there was no decrease in acute hypersensitivity among patients who received a single nefopam administration with no block. Therefore, to identify the effects of nefopam, further studies analyzing single drugs administered at various time points and dosages might be necessary.

There were no significant differences between the N and C groups in the use of postoperative rescue drugs (*p* = 0.187) (Table 5). The time of administration was before 48 h after surgery, regardless of ISB and postoperative rescue drugs were administered three times on average. This result did not prove that nefopam reduced opioid consumption compared with other studies. There was no difference in the total number of rescue drugs, indicating that the pain continued in the early postoperative period. This result suggests that nefopam might not be effective at controlling acute postoperative hyperalgesia occurring within 24 h regardless of the use of rescue drugs.

The following factors may have affected our study. First, this study examined pain from arthroscopic shoulder surgery and did not include patients with severe pain who underwent more major orthopedic interventions, such as spinal surgery or total hip or knee replacement, which are associated with VAS scores greater than 7. Among the patients who received ISB, the VAS score remained below 4 during the entire postoperative period. Even among patients not receiving nerve block, the VAS score had an average that was less than 7. In addition, 24 h after surgery, the VAS score was less than 4 regardless of the use of a nerve block. Second, although arthroscopic shoulder surgery causes the worst pain within 24 h after surgery, the restriction of motion due to the shoulder immobilizer, an aspect of postoperative management, results in less pain than usual and the pain continues intermittently with a similar pain severity for a long time. Thus, we cannot clearly ascertain the analgesic effect of nefopam in contrast to other orthopedic surgeries [19]. More definitive results might have been produced if we had applied other administration doses and methods.

The effects on PONV revealed that nefopam could reduce opioid consumption [6,20,21] and cause a consequent decrease in opioid-induced side effects [11]. In our study, most patients had more than three risk factors for PONV (Figure 4). However, both the individual risk factors and all risk factors did not influence the occurrence of PONV. In contrast to the usual effect of combination analgesics, the N group developed significantly more PONV (*p* = 0.023) (Table 8). We can predict that the neurotransmitters that act on nefopam (which are related to the analgesic effect) and the neurotransmitters related to nausea and vomiting might be different or play largely unrelated roles. For example, serotonin plays a key role in pain control mechanisms and acts through multiple serotonin receptor subtypes (5-HT1, 5-HT2 and 5-HT3 receptors), which are thought to be involved in the serotonin-mediated anti-nociceptive mechanism [22]. However, our finding of an increase in PONV showed that the 5-HT3 receptor antagonists related to nausea and vomiting among the subtypes had limited influence on nefopam. In other words, receptors that affect the anti-nociceptive mechanism and receptors have an effect on the function of PONV antagonists through different pathways. These findings also suggest that two of the other triple neurotransmitter uptake inhibitors can be applied in the same way. Therefore, the anti-nociceptive and antiemetic effects of nefopam on neurotransmitters should be studied in the near future.

In this study, there were no acute postoperative analgesic or anti-hyperalgesic effects [19] when nefopam was used as a non-opioid centrally acting inhibitor of serotonin, epinephrine and dopamine uptake at synapses. Nefopam is predicted to function as a delayed postoperative analgesic agent based on the VAS score 24 h after surgery. In addition, it has been difficult to demonstrate the preventive analgesic effects of nefopam by its early anti-nociceptive action as a single agent and it shows no benefit in decreasing PONV, one of the minor side effects [23]. This finding should be further investigated in terms of antiemetic and anti-nociceptive receptors.

There were some limitations of our study. First, there are limitations on the dose and usage of nefopam. In previous studies, nefopam was applied as a preoperative, intraoperative and postoperative continuous infusion through IV-PCA. In other words, using only the nefopam without opioids means that we are unable to prove the definite effect as an alternative or find a maximizing effect only through continuous administration. Since all patients were only given a limited dose for a short period of time, administering this low dose in the short term prevented us from demonstrating typical results. Further studies of the opioid-equivalent analgesic dosage and injection method will be needed. Second, in our orthopedics departments, rescue drugs are injected at regular time intervals whenever patients complain postoperative pain. Therefore, this does means that the patient’s pain status and evaluation are not directly measured by our rescue drug protocol. Therefore, PRNs and rescue drugs must be taken into account when considering objective indicators in future analysis.

## 5. Conclusions

Arthroscopic rotator cuff repair produces pain that is less severe than other open orthopedic surgeries but is accompanied by early, intense and long-lasting postoperative pain. Despite the non-opioid, centrally acting analgesic action of nefopam, there are currently no definitive answers regarding its anti-hyperalgesic and analgesic effects when applied as a single agent preemptively and immediately after surgery in a certain dose. This result might be expected to be influenced by the dose and application method of nefopam. To confirm the efficacy of nefopam, the studies in the near future should analyze various timely appropriate methods of administering nefopam as a single agent (not a combination) rather than the continuous intraoperative and postoperative infusion approach of previous studies.

## 6. The Clinical Study Registration Numbers

This study was approved by Institutional review board (IRB) (Registration number: KC15OISI0446) and Clinical Research information service (CRIS) (Registration number: KCT00023254).

## Figures and Tables

**Table 1:** Patient data description.

Variable	Total	Control Group	Nefopam Group	*p*-Value *
Total				
Age				
Mean ± SD	57.7 ± 13.8	57.2 ± 13.7	58.2 ± 13.9	0.348
median (range)	61 (18–82)	60 (18–82)	61.5 (19–82)	
Sex				
male	80 (44.4)	37 (46.2)	43 (53.8)	0.453
female	100 (55.6)	53 (53)	47 (47)	
Height				
Mean ± SD	1.6 ± 0.1	1.6 ± 0.1	1.6 ± 0.1	0.117
median (range)	1.62 (1.42–1.84)	1.6 (1.42–1.8)	1.62 (1.45–1.84)	
Weight				
Mean ± SD	64.8 ± 10.1	63.5 ± 9.2	66.1 ± 10.9	0.164
median (range)	64 (43.9–94)	63.5 (43.9–90)	65 (47–94)	
BMI				
Mean ± SD	25.7 ± 15.8	24.3 ± 3.9	27.1 ± 22	0.817
median (range)	24.56(2.46–231.01)	24.55 (2.46–32.8)	24.59 (18.07–231.01)	
Operative time				
Mean ± SD	87.6 ± 30.2	83.3 ± 28.6	91.9 ± 31.2	0.065
median (range)	85 (32–200)	75 (32–195)	90 (40–200)	
ASA				
1	77 (42.8)	39 (50.6)	38 (49.4)	>0.999
2	102 (56.7)	51 (50)	51 (50)	
3	1 (0.6)	0 (0)	1 (100)	
Block				
no	90 (50)	45 (50)	45 (50)	>0.999
yes	90 (50)	45 (50)	45 (50)	

Values are numbers (percentages) of categorical variables and other variables are shown as means (SD) or medians (range). Wilcoxon rank-sum test was used for continuous variables and the Chi-square test for categorical variables. Mean ± SD, mean ± standard deviation; BMI, body mass index; ASA-PS, American Society of Anesthesiologists physical status; *: *p* < 0.05.

**Table 2:** Association between nefopam and VAS at each time point.

Variable	Total	Control Group	Nefopam Group	*p*-Value *
T0 (preop)				
VAS				
Mean ± SD	2 ± 0.8	1.98 ± 0.79	2.02 ± 0.81	0.78
median (range)	2 (0–4)	2 (0–4)	2 (0–4)	
T1 (0)				
VAS				
Mean ± SD	3.33 ± 3.03	3.39 ± 3.22	3.28 ± 2.85	0.987
median (range)	2 (0–10)	2 (0–10)	2 (0–8)	
T2 (30 min)				
VAS				
Mean ± SD	2.83 ± 2.32	2.69 ± 2.24	2.97 ± 2.39	0.518
median (range)	2 (0–8)	2 (0–8)	2 (0–8)	
T3 (12 h)				
VAS				
Mean ± SD	3.44 ± 2	3.63 ± 2.13	3.24 ± 1.86	0.235
median (range)	3 (0–8)	3 (0–8)	3 (0–8)	
T4 (24 h)				
VAS				
Mean ± SD	2.88 ± 1.33	3.04 ± 1.36	2.72 ± 1.29	0.083
median (range)	3 (0–7)	3 (0–6)	3 (0–7)	
T5 (48 h)				
VAS				
Mean ± SD	2.34 ± 1.06	2.41 ± 1.07	2.28 ± 1.06	0.398
median (range)	2 (0–6)	2 (0–6)	2 (0–6)	

Wilcoxon rank-sum test; mean ± SD, mean ± standard deviation; VAS, visual analogue scale. T0: before surgery, T1: immediately after surgery, T2: after surgery 30 min; T3: after surgery 12 h; T4: after surgery 24 h; and T5: after surgery 48 h. *, *p* < 0.05.

**Table 3:** Association between sex and VAS at each time point.

	Control Group		Nefopam Group		*p*-Value	
	Male (*n* = 37)	Female (*n* = 53)	Male (*n* = 43)	Female (*n* = 47)	Male	Female
T0	2.03 ±0.76	1.92 ± 0.83	2.07 ± 0.67	1.98 ± 0.92	0.987	0.720
T1	3.32 ± 3.44	3.40 ± 3.12	2.72 ± 2.61	3.79 ± 2.99	0.740	0.582
T2	2.57 ± 2.43	2.77 ± 2.12	2.60 ± 2.18	3.30 ± 2.54	0.729	0.373
T3	3.68 ± 2.14	3.55 ± 2.20	2.86 ± 1.75	3.60 ± 1.90	0.068	0.837
T4	3.03 ± 1.66	3.03 ± 1.13	2.53 ± 1.32	2.89 ± 1.26	0.099	0.651
T5	2.27 ± 1.02	2.49 ± 1.10	2.21 ± 1.19	2.34 ± 0.93	0.616	0.629

T0: before surgery, T1: immediately after surgery, T2: after surgery 30 min; T3: after surgery 12 h; T4: after surgery 24 h; and T5: after surgery 48 h.

**Table 4:** Association in VAS between nefopam group and control group regarding block.

**No block**				
**Variable**	**Total**	**Control Group**	**Nefopam Group**	*p*-Value *
T 0 (preop)				
VAS				
mean ± SD	2 ± 0.73	2.02 ± 0.78	1.98 ± 0.69	0.942
median (range)	2 (0–4)	2 (0–4)	2 (0–3)	
T 1 (0)				
VAS				
mean ± SD	5.37 ± 2.6	5.6 ± 2.71	5.13 ± 2.49	0.326
median (range)	6 (0–10)	6 (0–10)	6 (0–8)	
T 2 (30 min)				
VAS				
mean ± SD	4.16 ± 2.09	4.09 ± 1.98	4.22 ± 2.22	0.906
median (range)	4 (1–8)	4 (2–8)	4 (1–8)	
T 3 (12 h)				
VAS				
mean ± SD	4.13 ± 1.63	4.49 ± 1.67	3.78 ± 1.52	0.058
median (range)	4 (0–8)	5 (2–8)	4 (0–8)	
T 4 (24 h)				
VAS				
mean ± SD	3.11 ± 1.18	3.18 ± 1.17	3.04 ± 1.19	0.85
median (range)	3 (0–6)	3 (1–6)	3 (0–5)	
T 5 (48 h)				
VAS				
mean ± SD	2.41 ± 1.09	2.36 ± 0.98	2.47 ± 1.19	0.656
median (range)	2 (0–6)	2 (1–5)	2 (0–6)	
**Block**				
**Variable**	**Total**	**Control Group**	**Nefopam Group**	*p*-Value *
T 0 (preop)				
VAS				
mean ± SD	2 ± 0.86	1.93 ± 0.81	2.07 ± 0.91	0.635
median (range)	2 (0–4)	2 (0–3)	2 (0–4)	
T 1 (0)				
VAS				
mean ± SD	1.3 ± 1.84	1.18 ± 1.91	1.42 ± 1.79	0.298
median (range)	0 (0–6)	0 (0–6)	1 (0–6)	
T 2 (30 min)				
VAS				
mean ± SD	1.5 ± 1.68	1.29 ± 1.5	1.71 ± 1.84	0.291
median (range)	1 (0–7)	1 (0–6)	1 (0–7)	
T 3 (12 h)				
VAS				
mean ± SD	2.74 ± 2.1	2.78 ± 2.2	2.71 ± 2.02	0.967
median (range)	3 (0–7)	3 (0–7)	3 (0–7)	
T 4 (24 h)				
VAS				
mean ± SD	2.66 ± 1.44	2.91 ± 1.52	2.4 ± 1.32	0.03 *
median (range)	2.5 (0–7)	3 (0–6)	2 (0–7)	
T 5 (48 h)				
VAS				
mean ± SD	2.28 ± 1.04	2.47 ± 1.16	2.09 ± 0.87	0.088
median (range)	2 (0–6)	2 (0–6)	2 (0–4)	

Wilcoxon rank-sum test: mean ± SD, mean ± standard deviation; VAS, visual analogue scale. *, *p* < 0.05.

**Table 5:** Association between nefopam and total number of PRNs.

	ITT		
Variable	Control Group	Nefopam Group	*p*-Value *
Total PRN during 48 h (time point T0–T5)			
mean ± SD	3.1 ± 2.2	3.6 ± 2.7	0.187
median (range)	3 (0–12)	3 (0–12)	

Wilcoxon rank-sum test; ITT, intention to treat mean ± SD, mean ± standard deviation; PRN, as required; *, *p* < 0.05.

**Table 6:** PONV risk factors associated with PONV.

	PONV			
Variable	Non Event	Event	OR (95% CI)	*p*-Value *
Smoking				
smoking	1 (94.7)	1 (5.3)	2.41 ^ǂ^	
nonsmoking	142 (88.2)	19 (11.8)	(0.3–19.08)	0.405
Sex				
male	65 (86.7)	10 (13.3)	0.68 ^ǂ^	
female	95 (90.5)	10 (9.5)	(0.27–1.74)	0.425
PONV history				
no	155 (89.6)	18 (10.4)	3.44 ^ǂ^	
yes	5 (71.4)	2 (28.6)	(0.62–19.06)	0.157
Postop opioid				
no	11 (78.6)	3 (21.4)	0.42 ^ǂ^	
yes	148 (89.7)	17 (10.3)	(0.11–1.66)	0.217

^ǂ^: Reference; Univariate logistic regression; OR, Odds Ratio; PONV, postoperative nausea and vomiting; CI, confidence interval; *, *p* < 0.05.

**Table 7:** PONV risk factors associated with PONV and nefopam.

**PONV No Event**			
**Variable**	**Control Group**	**Nefopam Group**	***p*-Value**
Smoking			
smoking	8 (44.4)	10 (55.6)	0.594
nonsmoking	77 (54.2)	65 (45.8)	
Sex			
male	32 (49.2)	33 (50.8)	0.512
female	53 (55.8)	42 (44.2)	
PONV Hx			
no	83 (53.5)	72 (46.5)	0.666
yes	2 (40)	3 (60)	
Postop opioid			
no	7 (63.6)	4 (36.4)	0.698
yes	78 (52.7)	70 (47.3)	
Operative time			
<60 min	17 (63)	10 (37)	0.362
≥60 min	68 (51.1)	65 (48.9)	
**PONV Event**			
**Variable**	**Control Group**	**Nefopam Group**	***p*-Value**
Smoking			
smoking	1 (100)	0 (0)	0.25
nonsmoking	4 (21.1)	15 (78.9)	
Sex			
male	2 (20)	8 (80)	>0.999
female	3 (30)	7 (70)	
PONV Hx			
no	4 (22.2)	14 (77.8)	0.447
yes	1 (50)	1 (50)	
Postop opioid			
no	1 (33.3)	2 (66.7)	>0.999
yes	4 (23.5)	13 (76.5)	
Operative time			
<60 min	1 (25)	3 (75)	>0.999
≥60 min	4 (25)	12 (75)	

Chi-square test or Fisher’s exact test; PONV, postoperative nausea and vomiting.

**Table 8:** Association between PONV and nefopam.

	PONV			
Variable (Nefopam)	No Event	Event	OR (95% CI)	*p*-Value *
no nefopam	85 (94.4)	5 (5.6)		
nefopam	75 (83.3)	15 (16.7)	3.4 (1.18–9.8)	0.023 *

Univariate logistic regression; CI, confidence interval; PONV, postoperative nausea and vomiting; *, *p* < 0.05.

**Table 9:** Association between PONV and total number of risk factors.

	PONV			
Variable	No Event	Event	OR (95% CI)	*p*-Value *
Total number of risk factors				
mean ± SD	2.45 ± 0.77	2.4 ± 0.82	0.93 (0.51–1.68)	0.799
median(range)	3 (0–4)	2.5 (1–4)		

Univariate logistic regression; mean ± SD, mean ± standard deviation. PONV, postoperative nausea and vomiting; *, *p* < 0.05.
